# Virulence Genes as Markers for *Pseudomonas aeruginosa* Biofilm Formation in Dogs and Cats

**DOI:** 10.3390/ani12040422

**Published:** 2022-02-10

**Authors:** Daria Płókarz, Michał Czopowicz, Karolina Bierowiec, Krzysztof Rypuła

**Affiliations:** 1 Division of Infectious Diseases of Animals and Veterinary Administration, Department of Epizootiology and Clinic of Birds and Exotic Animals, Faculty of Veterinary Medicine, Wroclaw University of Environmental and Life Sciences, pl. Grunwaldzki 45, 50366 Wroclaw, Poland; daria.plokarz@upwr.edu.pl (D.P.); karolina.bierowiec@upwr.edu.pl (K.B.); 2 Division of Veterinary Epidemiology and Economics, Institute of Veterinary Medicine, Warsaw University of Life Sciences-SGGW, Nowoursynowska 159c, 02776 Warsaw, Poland; mczopowicz@gmail.com

**Keywords:** biofilm, *Pseudomonas aeruginosa*, virulence genes, dogs, cats

## Abstract

**Simple Summary:**

*Pseudomonas aeruginosa* is an opportunistic pathogen of dogs and cats able to cause both local and systemic infections. This bacterium is widespread in the environment, resistant to unfavorable conditions, and may spread between humans and other mammals. Its virulence and transmission rely on various virulence factors including those responsible for biofilm formation. Biofilm is defined as a complex biological system that is composed of exopolysaccharides, proteins, extracellular DNA, and biomolecules. Extracellular polymeric substances are the main ingredients of biofilm, accounting for 90% of its total biomass. In this study we analyzed the prevalence of five virulence genes involved in biofilm formation (pelA, pslA, ppyR, fliC and nan1) in 271 *P. aeruginosa* isolates obtained from dogs and cats. All animals had clinical symptoms of *P. aeruginosa* infection. In dogs, the strains were isolated from the external auditory canal, respiratory tract, and skin. In cats, the strains were isolated from the nasal cavity, external auditory canal, and skin. Biofilm-forming strains accounted for 90.6% of *P. aeruginosa* isolates from dogs and 86.4% from cats. The most commonly identified virulence factor gene was ppyR (97.4%). The fliC and pslA genes were detected in 62.4% and 60.1% of the study population, respectively, whereas nan1 and pelA genes were found in 45.0% and 38.7%, respectively. Prevalence of the virulence factor genes was not significantly different between dogs and cats. Given that the ability to form biofilm is related to the antibiotic resistance of *P. aeruginosa*, our results indicate potential candidates for biomarkers assisting in selection of the most effective treatment for *P. aeruginosa* infections.

**Abstract:**

*Pseudomonas aeruginosa* is an ubiquitous bacterium and opportunistic pathogen that plays an important role in nosocomial infections. The presence of virulence factors and the biofilm-forming ability of this species contributes to a high risk of treatment complications. In this study, we examined the biofilm-forming ability and the prevalence of five virulence factor genes (pslA, pelA, ppyR, fliC, and nan1) in 271 *P. aeruginosa* isolates (212 from dogs and 59 from cats). Biofilm-forming ability was detected in 90.6% of isolates in dogs and 86.4% of isolates in cats. In *P. aeruginosa* isolates from both species, the most prevalent virulence factor gene was ppyR (97.2% in dogs and 98.3% in cats), followed by pslA (60.8% and 57.6%), fliC (60.4% and 69.5%), nan1 (45.3% and 44.1%), and pelA (40.1% and 33.9%, respectively). In dogs, a significantly higher proportion of biofilm-forming *P. aeruginosa* strains possessed the fliC gene compared to non-biofilm-forming strains (*p* = 0.015). In cats, a significantly lower proportion of biofilm-forming strains had the nan1 gene compared to non-biofilm-forming strains (*p* = 0.017). In conclusion, the presence of fliC gene and the absence of nan1 gene could be indicators of biofilm-forming ability of *P. aeruginosa*.

## 1. Introduction

*Pseudomonas aeruginosa* (*P. aeruginosa*) is a ubiquitous Gram-negative bacillus. It is also an opportunistic pathogen that occurs on the skin and mucosal membranes of humans and other mammals. In dogs and cats, *P. aeruginosa* causes skin, systemic and urinary tract infections [1], as well as ulcers, hemorrhagic crusts, erythematous papules [2], otitis externa [3], conjunctivitis [4], rhinosinusitis [5], periapical tooth abscesses [6], and periodontal disease [7]. These infections are consistent with the “One Health” concept, because of the bacterium persistence in the environment, and possible transmission between humans and other mammals.

Biofilm is defined as a complex biological system that is composed of exopolysaccharides, proteins, extracellular DNA, and biomolecules [8]. Extracellular polymeric substances (EPS) are the main ingredients of biofilm, forming 50–90% of total biofilm biomass [3]. *P. aeruginosa* with biofilm-forming ability colonize both biotic and abiotic surfaces. The increasing number of experiments on this subject confirm that chronic infection is caused by bacterial biofilm formation, rather than the planktonic form of bacteria, which causes acute infection [9]. The quorum-sensing system controls the ability of flagellated microorganisms to transform from the planktonic into the biofilm form [8].

The various mechanisms involved in biofilm formation are related to the production of virulence factors, which are encoded by established genes. Crucial components of the biofilm structure are the exopolysaccharide containing components Psl and Pel. Pel is required to form biofilm on solid surfaces; on the other hand, Psl regulates adhesion to solid surfaces and initiates biofilm growth by developing new microcolonies [10,11]. The pslA and pelA genes participate in the formation of the carbohydrate-rich structure of the biofilm matrix. Moreover, after inactivation of the ppyR gene, the Psl operon is suppressed and a decline in biofilm formation occurs. The ppyR gene encodes a putative transmembrane protein. The fliC gene is a crucial component of flagella production, due to subunit protein-encoding-flagellin type B [12]. Flagella are highly immunogenic, and are essential for inflammation development [13]. Flagellin *P. aeruginosa* strains releases epithelial TLR5 signaling NF-kB. Flagellum mediated-swimming is one of several types of motilities in *P. aeruginosa* strains. The mutants of *P. aeruginosa* that do not have flagella have poor colonization ability [12].

Another virulence factor is neuraminidase, which is an extracellular factor involved in the implantation of *P. aeruginosa*. Neuraminidase may upregulate a number of potential bacterial receptors and it might release terminal sialic acid residues from sialylated gangliosides [14,15]. Wolska et al. [16] obtained a statistically significant difference in the adhesion of buccal epithelial cells in *P. aeruginosa* strains that contained one neuraminidase encoding gene (nan1) versus strains in which nan1 was not detected.

In this study we aimed to analyze the prevalence of five virulence factor genes (pelA, pslA, ppyR, fliC, and nan1) that could affect biofilm formation in *P. aeruginosa* strains from dogs and cats.

## 2. Materials and Methods

This study included 271 isolates collected from dogs (*n* = 212) and cats (*n* = 59) in the Lower Silesia, Poland, between 2017 and 2020. Male-to-female ratio was close to 1. The age of animals ranged from 2 months to 18 years (median of 7 years) and was significantly higher in dogs than in cats (*p* = 0.001). Pedigree individuals accounted for 80% of dogs, whereas the domestic shorthair breed predominated among cats (Table 1). All animals had clinical symptoms of *P. aeruginosa* infection, which was confirmed in microbiological tests. In dogs, the external auditory canal was the most common collection site, followed by the respiratory system, and skin. In cats, most of *P. aeruginosa* strains were isolated from the nasal cavity. These strains were significantly more often isolated from the external auditory canal, skin, and appendages of dogs, and from the respiratory tract of cats (Table 1).

### 2.1. Isolates

The isolates of non-fermentative gram-negative bacilli were transferred to basic media. All isolates were cultured on a Columbia Blood Agar Base Thermo Scientific OXOID with 5% sheep blood and MacConkey Agar (Thermo Scientific OXOID, Gdańsk, Poland). The isolates were then checked for oxidase production (Oxidase Detection Strips MICROGEN MID-61g GRASO BIOTECH, Starogard Gdański, Poland). Isolates were frozen with Brain Heart Infusion Broth (BHI) (Thermo Scientific OXOID, Gdańsk, Poland) supplemented with 30% glycerol.

### 2.2. DNA Extraction

Oxidase positive isolates were cultured on a Columbia Blood Agar Base with 5% sheep blood (Thermo Scientific OXOID, Gdańsk, Poland) and were incubated overnight at 37 °C. A few colonies from each strain were added to 200 μL distilled water, shaken, and boiled for 20 min. The samples were then frozen at −20 °C for 5 min. After deactivation, the suspensions were centrifuged at 13,000 rpm for 3 min. The supernatant was preserved as template DNA.

### 2.3. PCR Assay

After pre-selection, isolates with *P. aeruginosa* identification were confirmed by polymerase chain reaction (PCR) based on amplification of two outer membrane lipoproteins genes, oprI and oprL. OprL encodes lipoprotein specific for *P. aeruginosa*, and oprI encodes lipoprotein which occur in both other fluorescent pseudomonads and *P. aeruginosa* [17].

The DNA was amplified in a thermocycler (Bio-Rad, Marnes-la-Coquette, France), using modified methods. The methods and primers are presented in Table 2.

Genes were amplified using 2.5 µL buffer (MgCl_2_ at 20 mM concentration), 0.2 µL Taq DNA Polymerase (5 U/L) (Thermo Fischer Scientific, Vilnius, Lithuania), 0.2 µL dNTP Mix 10 (Thermo Fischer Scientific, Vilnus, Lithuania), 0.2 µL of each specific primer created by Genomed S.A. (Warsaw, Poland) (Table 2), and 2 µL template DNA. The solution was increased to a 25 µL volume with sterile water.

Electrophoresis was performed in 2% Agarose gel with Midori Green (NIPPON Genetics EUROPE, Düren, Germany), and Marker 2 (A&A Biotechnology, Gdańsk, Poland) was used as a DNA ladder and detected by UV transillumination (Bio-Rad, Marnes-la-Coquette, France). Genetic material of *P. aeruginosa* ATCC 27853 strain was used as a positive control, and Mili Q water as a negative control.

### 2.4. Microtiter Plate Method

The microtiter plate method (MTP) was performed following the method of O’Toole et al. [20]. Pure colonies of strains were cultured on Columbia Agar with 5% sheep blood, after overnight incubation at 37 °C. A few colonies were diluted in a BHI broth, and incubated at 37 ℃ overnight. This culture was made equal with 0.5 McFarland standard, and was then diluted at a ratio of 1:100 in fresh a BHI medium. Then, 200 µL of the solution was added to a 96-well sterile microtiter plate. Each sample of *P. aeruginosa* isolate was replicated in eight wells. The fresh BHI broth was used as a negative quality control. Plates were incubated for 24 h at 37 °C. Then, the plate was turned over and the broth was shaken out. Next, water was gently added to each well and shaken out. This protocol was repeated twice. Then, 250 µL of a 0.1% solution of Cristal Violet, was added to each well. The microtiter plate was incubated at room temperature for 10 min, and was then washed three times by submerging it in a tube of water, and shaking it. The plate was left upside down for a few hours to dry completely [20]. Biofilm was quantified using the modified MTP method [21]. To each well, 250 µL of 95% ethanol was added to solubilize the crystal violet. Absorbance was measured (Spark 10M) at 590 nm. Sterile BHI was used as a negative control. *P. aeruginosa* ATCC 27853 with the capacity to form biofilm was used as a positive control. Biofilm-formation ability was considered as positive at a cut-off level 0.269. We determined cut-off arbitrarily by the mean for the negative control (culture medium, 0.149) plus two standard deviations (0.06). Levels of biofilm production were established based on following classification criteria: weak biofilm formers: 0.269 < A590 < 0.538 (2 × negative controls); moderate biofilm formers: 0.538 < A590 < 1.076 (4 × negative controls); strong biofilm formers: 1.076 < A590 (6 × negative controls).

### 2.5. Statistical Analysis

Categorical variables were expressed as counts in groups and percentages from the study population and compared between groups using the maximum likelihood G-test or Fisher’s exact test if an expected count in any cell of the contingency table was below 5. Proportions were compared between ordinal classes (strength of biofilm-forming ability) using the χ^2^ test for trends [22]. The 95% confidence interval (CI 95%) for proportions (prevalence) was calculated using the Wilson score method [23]. Numerical variables were presented as the median, interquartile range (IQR), and range, and compared between groups using the Mann–Whitney U test. The same test was used to compare ordinal variables (strength of biofilm-forming ability) between groups. Correlations between ordinal and numerical variables were analyzed using the Spearman’s rank correlation coefficient (R_s_). All tests were two-tailed. A significance level (α) was set at 0.05. Statistical analysis was performed using TIBCO Statistica 13.3 (TIBCO Software Inc., Palo Alto, CA, USA).

## 3. Results

**Figure 1:**
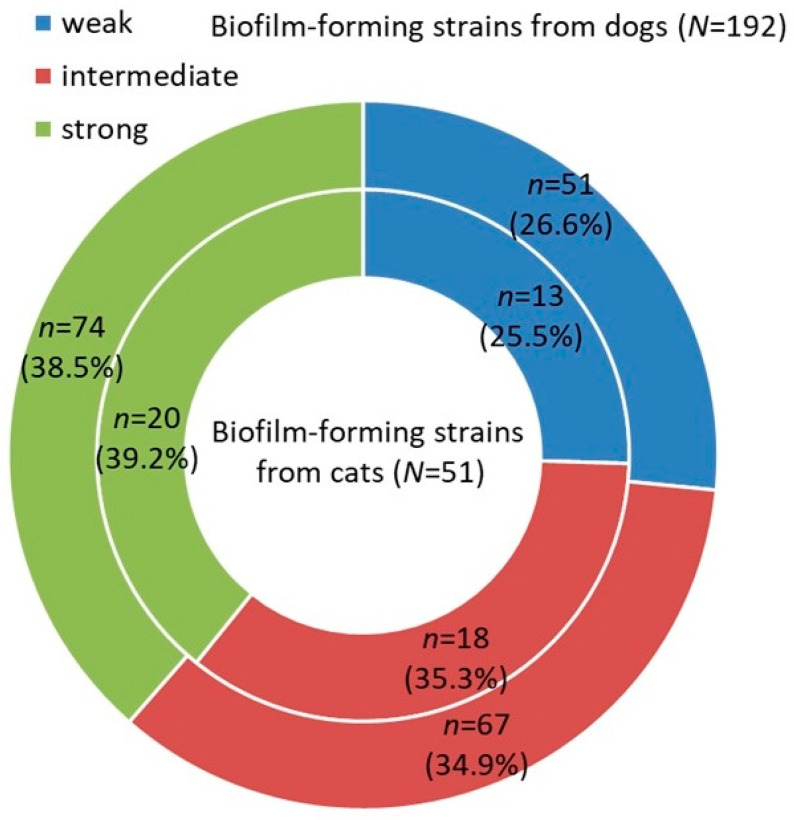
Proportion of biofilm-forming strains with various strength of biofilm-forming ability (weak, intermediate, and strong) isolated from dogs (outer cycle) and cats (inner cycle).

Biofilm-forming strains accounted for 90.6% (CI 95%: 85.9%–93.8%; *n* = 192) of *P. aeruginosa* isolates from dogs, and 86.4% (CI 95%: 75.5%–93.0%; *n* = 51) of *P. aeruginosa* isolates from cats. Proportions of biofilm-forming strains with weak (*n* = 64; 26.3% of 243 biofilm-forming strains), intermediate (*n* = 85; 35.0%), and strong (*n* = 94; 38.7%) biofilm-forming ability were similar, and their distribution was not significantly different between dogs and cats (*p* = 0.893) (Figure 1).

The prevalence of biofilm-forming strains did not significantly differ between males and females (dogs: 90.2% vs. 91.0%, respectively, *p* = 0.838; cats: 91.2% vs. 80.0%, respectively, *p* = 0.265) or among collection sites either in dogs (*p* = 0.092) or in cats (*p* = 0.999) (Table 3).

The strength of biofilm-forming ability was neither different between sexes (*p* = 0.731 in dogs and *p* = 0.934 in cats) nor correlated with animals’ age either in dogs (R_s_ = 0.11; *p* = 0.148) or in cats (R_s_ = 0.29; *p* = 0.059).

**Figure 2:**
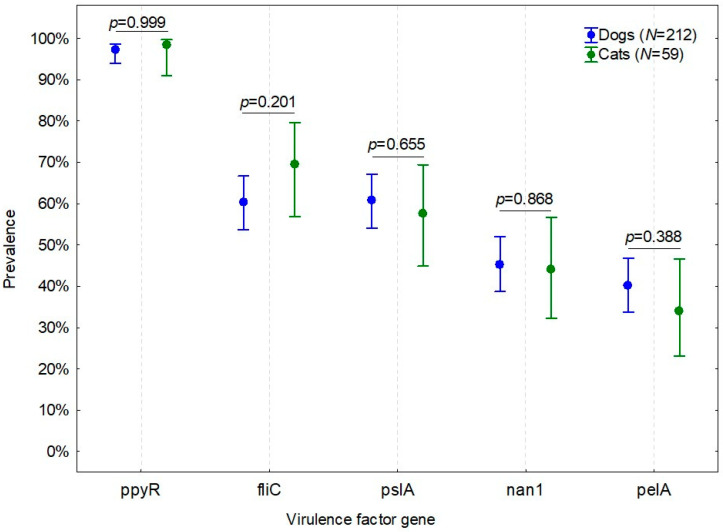
Prevalence (CI 95%) of virulence factor genes in *P. aeruginosa* strains isolated from dogs and cats.

The most commonly identified virulence factor gene was ppyR (*n* = 264; 97.4%, CI 95%: 94.8%–98.7%). The fliC (*n* = 169; 62.4%, CI 95%: 56.5%–67.9%) and pslA (*n* = 163; 60.1%, CI 95%: 54.2%–65.8%) genes were detected significantly less often, whereas nan1 (*n* = 122; 45.0%, CI 95%: 39.2%–51.0%) and pelA (*n* = 105; 38.7%, CI 95%: 33.1%–44.7%) genes were observed in the lowest proportion of isolates. Prevalence of the five virulence factor genes was not significantly different between dogs and cats (Figure 2).

The only virulence factor genes whose prevalence significantly differed between biofilm-forming and non-biofilm-forming *P. aeruginosa* strains were the fliC gene in dogs (significantly more often detected in biofilm-forming *P. aeruginosa* strains; *p* = 0.015) and nan1 gene in cats (significantly less often detected in biofilm-forming *P. aeruginosa* strains; *p* = 0.017) (Table 4).

**Figure 3:**
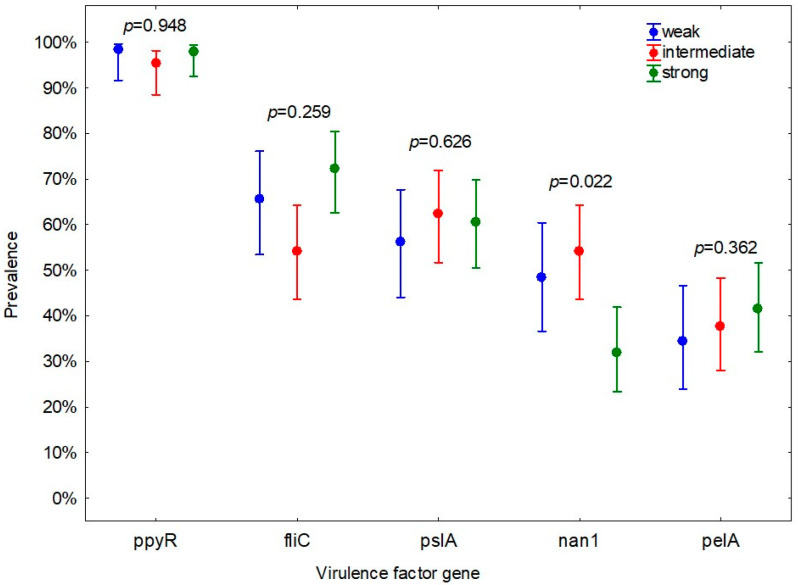
Prevalence (CI 95%) of virulence factor genes in biofilm-forming *Pseudomonas aeruginosa* strains with weak (*n* = 64), intermediate (*n* = 85), and strong (*n* = 94) biofilm-forming ability.

Only the prevalence of nan1 gene appeared to be significantly linked to the strength of biofilm-forming ability. A significantly lower proportion of strains with strong biofilm-forming ability than strains with weak and moderate biofilm-forming ability had nan1 gene (*p* = 0.022) (Figure 3).

## 4. Discussion

Biofilm formation is the most important virulence factor of *P. aeruginosa* and is often responsible for failures of the antibiotic treatment. Antibiotic resistance is higher among biofilm-forming strains compared to strains without this property. Commercial methods for testing antibiotic susceptibility rely on the minimum inhibitory concentration (MIC). However, this approach is only feasible for the planktonic phase of bacteria. Consequently, if clinicians do not know which phase of bacteria (planktonic versus biofilm-forming) is being examined, MIC indication may be misleading. Proper selection of antibiotic therapy in *P. aeruginosa* infection with the strain bearing virulence factor requires the minimum biofilm eliminating concentration (MBEC) be determined or additional biofilm removing substance be applied [24]. Analysis of cases with biofilm-forming *Pseudomonas* spp. strains shows that the MBEC should be determined [24,25]. Microbiological methods verifying *P. aeruginosa* biofilm-forming ability are time-consuming and require specific equipment and trained laboratory staff. Our study aimed at finding an easier and faster way to confirm *P. aeruginosa* biofilm-forming ability. We analyzed genes which could be related to biofilm forming based on the available literature.

Our study showed that only two virulence factor genes (fliC in canine strains, and nan1 in feline strains) could be linked to the biofilm-forming ability. The remaining genes occurred similarly often in both types of *P. aeruginosa* strains—the ppyR gene in virtually all examined strains, fliC and pslA in slightly more than a half, whereas nan1 and pelA were in less than a half of examined strains.

Milivojevic et al. [26] and Sharma et al. [5] previously showed that *P. aeruginosa* isolates had biofilm forming ability in 93% and 89% of dogs, respectively. This phenomenon was supported by our study, in which 86% and 91% of isolates in cats and dogs were biofilm producers, respectively. In our study, 34.9% and 33.9% of isolates in dogs and cats, respectively, were strong biofilm-producers. However, we are not able to compare the strength of biofilm-forming ability with our research because Milivojevic et al. [26] used PAO1 as a cut-off value of the MTP test, while we established the cut-off value arbitrarily. Results of biofilm-forming ability in humans and animals in the study by the mentioned authors were similar. This conclusion supports the necessity of controlling the occurrence and biofilm-forming ability of *P. aeruginosa* in companion animals.

In contrast to our study, Pye et al. [27] classified only 40% of *P. aeruginosa* samples isolated from dogs as biofilm formers. This discrepancy with our results might be attributed to the use of lysogeny broth (LB) medium. Wijesinghe et al. [28] showed that, compared to LB medium, BHI performed better at analyzing in vitro biofilm growth. A difference between Pye et al. [27] and our results may be associated with the period of time when the studies were carried out as the genetic content of *P. aeruginosa* strains collected could be different between the two studies.

The presence of pelA, pslA, ppyR, fliC, and nan1 genes has been previously associated with biofilm formation in human medicine [18,19,26]. However, the fact that they are also detectable in isolates from companion animals implies that they are universal. Samad et al. [29] obtained a similar prevalence for the pelA gene (44%) to our study, with 40% of *P. aeruginosa* samples from dogs harboring it. Higher prevalence might be associated with the origin of collected samples because Samad et al. [29] collected both clinical and environmental samples. In our study, only samples from companion animals were collected.

Ertugrul et al. [30] detected fliC gene in 60% of samples, and this result is similar to ours. We detected fliC in the *P. aeruginosa* samples of 60% and 70% of dogs and cats, respectively. We showed that the detection of two genes in dogs and cats could be used to determine *P. aeruginosa* biofilm-forming ability. The presence of fliC in dogs and the absence of nan1 in cats was significantly associated with biofilm formation. This latter finding could result from unknown elements in the quorum sensing mechanism for *P. aeruginosa* infection in cats. It could be related to nan1 gene occurrence and the planktonic form of *P. aeruginosa* that is responsible for acute infection. Lanotte et al. [14] showed that, although not statistically significant, the prevalence of the nan1 gene rose in cases with worse clinical status. The authors detected nan1 in the *P. aeruginosa* strain of 57% of patients with cystic fibrosis with good and excellent clinical status, and in 71% of patients with poor or weak clinical status.

Our study showed that cats were closer to the cystic fibrosis human model compared to dogs, because 83% of strains originated from the respiratory tract, compared to only 17% for dogs. We compared our results regarding nan1 and fliC genes with human data because no studies on these genes in companion animals have so far been published. To our knowledge, this study is the first to report the detection of nan1 and fliC genes in dogs and cats. However, our results on cats are at odds with the result obtained by Soong et al. [15] in humans, as a positive correlation between biofilm formation and neuraminidase production was observed in this study. This result might be attributed to the contribution of initial colonization in the airway. Thus, different neuraminidase might be produced under genetic control in particular species.

## 5. Conclusions

Our study indicates that analysis of *P. aeruginosa* biofilm-forming ability may be based on detection of selected virulence factor genes. The development of a simple marker for quick PCR gene detection could help support the treatment of *P. aeruginosa* infections both in human and animal medicine. It has to be stressed that this is a preliminary study, and further investigations and analyses are essential to introduce these results into medical practice.

## Figures and Tables

**Table 1:** Demographic characteristics of the study population and collection sites of *P. aeruginosa* strains isolated from dogs and cats.

Characteristic	Overall (*n* = 271)	Dogs (*n* = 212)	Cats (*n* = 59)	*p*-Value
Demographic Characteristics				
Age ^a^ (years)	7, 4–10 (0.2–18)	7, 4–10 (0.7–18)	5, 1–8 (0.2–16)	0.001 *
Male sex (n (%))	146 (53.9)	112 (52.8)	34 (57.6)	0.513
Breed (n (%))				<0.001 *
Crossbreed/Domestic shorthair	-	43 (20.3)	43 (72.9)	
Pedigree	-	169 (79.7)	16 (27.1)	
German Shepherd	-	33	-	
Cocker spaniel	-	25	-	
Beagle	-	12	-	
Golden retriever	-	10	-	
Shih-tzu	-	10	-	
Others	-	79	-	
Collection site				
External auditory canal	121 (44.6)	118 (55.7)	3 (5.1)	<0.001 *
Respiratory system and oral cavity	85 (31.4)	36 (17.0)	49 (83.1)	<0.001 *
Nasal cavity	51	7	44	
Conjunctival sac	12	10	2	
Throat	10	8	2	
Trachea and bronchi	7	6	1	
Oral cavity	5	5	0	
Skin and appendages	33 (12.2)	31 (14.6)	2 (3.4)	0.020 *
Skin	27	25	2	
Perianal glands	6	6	0	
Wounds	20 (7.4)	16 (7.5)	4 (6.8)	0.855
Genito-urinary tract	11 (4.1)	10 (4.7)	1 (1.7)	0.301
Vagina	7	7	0	
Urine	4	3	1	
Joint fluid	1 (0.4)	1 (0.5)	0	0.586

^a^ data available for 173 dogs and 43 cats; presented as the median, interquartile range, and range; * significant at α = 0.05.

**Table 2:** PCR assay reaction conditions and primers used in this study.

Target Genes	Primer Sequence 5′-3′	Product Size (bp)	Reference
pslA	F: 5′-TCCCTACCTCAGCAGCAAGC-3′ R: 5′–TGTTGTAGCCGTAGCGTTTCTG-3′	656	Ghadaksaz et al., 2015 [18]
pelA	F: 5′-CATACCTTCAGCCATCCGTTCTTC-3′ R: 5′-CGCATTCGCCGCACTCAG-3′	786	Ghadaksaz et al., 2015 [18]
ppyR	F: 5′-CGTGATCGCCGCCTATTTCC-3′ R: 5′-ACAGCAGACCTCCCAACCG-3′	160	Ghadaksaz et al., 2015 [18]
fliC	F: 5′-TGAACGTGGCTACCAAGAACG-3′ R: 5′-TCTGCAGTTGCTTCACTTCGC-3′	180	Immani et al., 2009 [19]
nan1	F: 5′-ACGCTCCGTCCAGCCGGA-3′ R: 5′-GTCTGGACGACGGCGGCA-3′	221	Lanotte et al., 2004 [14]
oprI	F: 5′ATGAACAACGTTCTGAAATTCTCTGCT-3′ R: 5′-CTTGCGGCTGGCTTTTTCCAG-3′	249	De Vos et al., 1997 [17]
oprL	F: 5′-ATGGAAATGCTGAAATTCGGC-3′ R: 5′-CTTCTTCAGCTCGACGCGACG-3′	504	De Vos et al., 1997 [17]

**Table 3:** Biofilm-forming *Pseudomonas aeruginosa* strains isolated from various collection sites.

Collection Site	Biofilm-Forming *P. aeruginosa* Strains	
	No. of Strains	Prevalence (CI 95%)
Dogs (*n* = 212)		
External auditory canal (*n* = 118)	111	94.1 (88.3–97.1)
Skin and appendages (*n* = 31)	28	90.3 (75.1–96.7)
Respiratory system and oral cavity (*n* = 36)	28	77.8 (61.9–88.3)
Genito-urinary tract (*n* = 10)	10	100 (72.2–100)
Wounds (*n* = 16)	14	87.5 (64.0–96.5)
Cats (*n* = 59)		
Respiratory system (*n* = 49)	42	85.7 (73.3–92.9)
Others (*n* = 10)	9	90.0 (59.6–98.2)

**Table 4:** Prevalence of virulence factor genes in 192 biofilm-forming and 20 non-biofilm-forming *P. aeruginosa* strains from dogs, and 51 biofilm-forming and 8 non-biofilm-forming *P. aeruginosa* strains from cats.

Virulence Factor Gene	*Pseudomonas aeruginosa* Strains				*p*-Value
	Biofilm-Forming		Non-Biofilm-Forming		
	*n*	Prevalence (CI 95%)	*n*	Prevalence (CI 95%)	
Dogs					
pslA	117	60.9 (53.9–67.6)	12	60.0 (38.7–78.1)	0.935
pelA	76	39.6 (32.9–46.6)	9	45.0 (25.8–65.8)	0.638
ppyR	186	96.9 (93.4–98.6)	20	100 (83.9–100)	0.999
fliC	121	63.0 (56.0–69.5)	7	35.0 (18.1–56.7)	0.015 *
nan1	88	45.8 (38.9–52.9)	8	40.0 (21.9–61.3)	0.617
Cats					
pslA	29	56.9 (43.3–69.5)	5	62.5 (30.6–86.3)	0.999
pelA	17	33.3 (22.0–47.0)	3	37.5 (13.7–69.4)	0.999
ppyR	50	98.0 (89.7–99.7)	8	100 (67.6–100)	0.999
fliC	35	68.6 (55.0–79.7)	6	75.0 (40.9–92.9)	0.999
nan1	19	37.3 (25.3–51.0)	7	87.5 (52.9–97.8)	0.017 *

* significant at α = 0.05.

## Data Availability

The datasets used and/or analyzed during the current study are available via e-mail from the corresponding author on reasonable request.
